# Development and validation of a nomogram to predict plastic bronchitis in children with refractory *Mycoplasma pneumoniae* pneumonia

**DOI:** 10.1186/s12890-022-02047-2

**Published:** 2022-06-27

**Authors:** Lihua Zhao, Tongqiang Zhang, Xiaojian Cui, Linsheng Zhao, Jiafeng Zheng, Jing Ning, Yongsheng Xu, Chunquan Cai

**Affiliations:** 1 Department of Respiratory, Tianjin Children’s Hospital (Children’s Hospital of Tianjin University), Tianjin, 300134 China; 2 Graduate School of Tianjin Medical University, Tianjin, 300134 China; 3 Department of Clinical Lab, Tianjin Children’s Hospital (Children’s Hospital of Tianjin University), Tianjin, 300134 China; 4 Department of Pathology, Tianjin Children’s Hospital (Children’s Hospital of Tianjin University), Tianjin, 300134 China; 5 Tianjin Pediatric Research Institute, Tianjin Children’s Hospital (Children’s Hospital of Tianjin University), Tianjin Key Laboratory of Birth Defects for Prevention and Treatment, Tianjin, 300134 China

**Keywords:** Plastic bronchitis, Refractory *Mycoplasma pneumoniae* pneumonia, LASSO, Nomogram, Risk factor

## Abstract

**Background:**

Early identification of plastic bronchitis (PB) is of great importance and may aid in delivering appropriate treatment. This study aimed to develop and validate a nomogram for predicting PB in patients with refractory *Mycoplasma pneumoniae* pneumonia (RMPP).

**Methods:**

A total of 547 consecutive children with RMPP who underwent fiberoptic bronchoscopy (FOB) intervention from January 2016 to June 2021 were enrolled in this study. Subsequently, 374 RMPP children (PB: 137, without PB: 237) from January 2016 to December 2019 were assigned to the development dataset to construct the nomogram to predict PB and 173 RMPP children from January 2020 to June 2021 were assigned to the validation dataset. The clinical, laboratory and radiological findings were screened using Least Absolute Shrinkage and Selection Operator (LASSO) regression and logistic regression was applied to construct a nomogram. The performance of the nomogram was evaluated by discrimination, calibration and clinical utility. Comparsion of ROC analysis and decision curve analysis (DCA) between nomogram and other models was performed to evaluate the discrimination ability and clinical utility.

**Results:**

The development dataset included 374 patients with a mean age of 6.6 years and 185(49.5%) were men. The validation dataset included 173 patients and the mean age of the dataset was 6.7 years and 86 (49.7%) were men. From 26 potential predictors, LASSO regression identified 6 variables as significant predictive factors to construct the nomogram for predicting PB, including peak body temperature, neutrophil ratio (N%), platelet counts (PLT), interleukin-6 (IL-6), actic dehydrogenase (LDH) and pulmonary atelectasis. The nomogram showed good discrimination, calibration and clinical value. The mean AUC of the nomogram was 0.813 (95% CI 0.769–0.856) in the development dataset and 0.895 (95% CI 0.847–0.943) in the validation dataset. Through calibration plot and Hosmer–Lemeshow test, the predicted probability had a good consistency with actual probability both in the development dataset (*P* = 0.217) and validation dataset (*P* = 0.183), and DCA showed good clinical utility. ROC analysis indicated that the nomogram showed better discrimination ability compared with model of peak body temperature + pulmonary atelactsis and another model of N% + PLT + IL-6 + LDH, both in development dataset (AUC 0.813 vs 0.757 vs 0.754) and validation dataset (AUC 0.895 vs 0.789 vs 0.842).

**Conclusions:**

In this study, a nomogram for predicting PB among RMPP patients was developed and validated. It performs well on discrimination ability, calibration ability and clinical value and may have the potential for the early identification of PB that will help physicians take timely intervention and appropriate management.

**Supplementary Information:**

The online version contains supplementary material available at 10.1186/s12890-022-02047-2.

## Background

*Mycoplasma pneumoniae* (MP) is a significant pathogen of community-acquired pneumonia (CAP) occurring primarily in children and young adults. MP pneumonia (MPP) accounts for 10–40% of CAP [1, 2], and is generally considered to be self-limited and benign. However, some cases may progress to refractory MPP (RMPP) despite appropriate administration of macrolides for 7 days or even longer [3, 4], which manifest as deterioration in clinical manifestations and radiological findings and often leads to longer disease course and various complications. Although the underlying mechanisms of RMPP are still uncertain, previous studies have identified that the excessive immunological inflammation play an important role in the development of RMPP, providing a theoretical basis for the application of glucocorticoids in the treatment of RMPP [5–8]. Glucocorticoids have been confirmed had a promising efficacy in alleviating the immune reaction and promoting recovery of RMPP [5, 9]. However, some cases with RMPP manifested as unresponsiveness to standard glucocorticoids and often required higher dose of glucocorticoids and further investigation of Fiberoptic bronchoscopy (FOB) therapy. Our previous study found that compared with glucocorticoids sensitive patients, this kind of RMPP patients had a higher incidence of mucus plug formation and plastic bronchitis (PB) [5]. The development of PB may be an important factor to make MPP refractory.

PB is an acute and critical pulmonary disease which is characterized by formation of bronchial casts(BCs) which can partially or completely obstruct the tracheobronchial tree [10]. The clinical manifestations of PB caused by infection include repeated fever, shortness of breath and can rapidly progress to acute dyspnea and respiratory failure, even life-threatening respiratory and circulatory failure [11, 12]. Through direct clearance of BCs in the airway, FOB and bronchoalveolar lavage (BAL) procedure is of prominent efficacy in treatment of PB. Therefore, early identification of likely BCs formation and development of PB that will require FOB and BAL therapy is of great importance.

This study aims to construct a risk prediction model based on clinical manifestations, laboratory blood indicators and radiological findings to help clinicians identify patients who are at high risk of PB among children with RMPP.

## Subjects and methods

### Study population

**Fig. 1:**
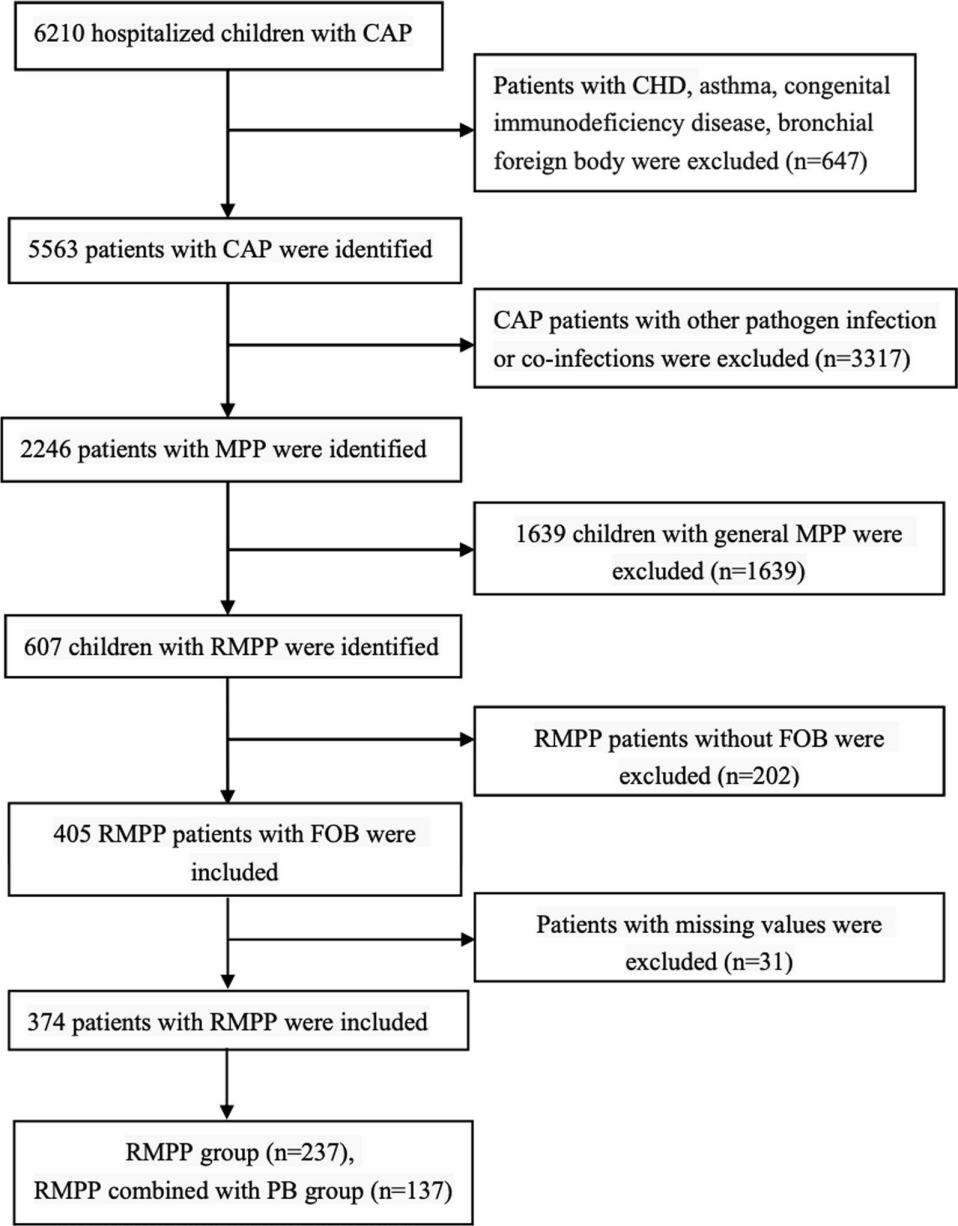
Study flow. *CAP* community-acquired pneumonia, *CHD* congenital heart disease, *MPP* *Mycoplasma pneumoniae* pneumonia, *PB* plastic bronchitis

This study was approved by the ethics committee of the Tianjin Children’s Hospital and conducted in accordance with the Declaration of Helsinki guidelines. The written informed consent was waived owing to the retrospective design of the study. A flowchart of our research is provided in Fig. 1. Exclusion criteria: (1) patients who had underlying disease, such us congenital heart disease, asthma and congenital immunodeficiency disease. (2) patients who had history of inhalation of foreign body and confirmed by FOB as bronchial foreign body. (3) Patients co-infected with other pathogens and tuberculosis. (4) patients who had incomplete medical records. Medical records from 374 RMPP patients who received fiberoptic bronchoscopy (FOB) and bronchoalveolar lavage (BAL) therapy were analyzed as the development dataset between January 2016 and December 2019 retrospectively. The 374 RMPP patients were divided into 2 groups based on the manifestations of bronchoscopy and histopathology: the RMPP group (237) and RMPP combined with PB group (137).

MPP was diagnosed based on followings [13]: (1) symptoms and signs indicative of pneumonia, including fever, cough and abnormal lung auscultation; (2) a new infiltration on chest radiograph; (3) positive laboratory results for MP, including an MP-immunoglobulin M (IgM) titer ≥ 1:160 or four-fold rising titer in acute and convalescent serum specimens; positive results for MP polymerase chain reaction (PCR) tests in the fluid of BAL or pleural effusion. RMPP was defined as follows [14]: diagnosed with MPP and no improvement or even deterioration in clinical manifestations and chest radiographical findings after appropriate administration of macrolides for 7 days. Someone would undergo the CT scan if he or she had one of the following conditions: (1) An inconsistency between clinical manifestations and chest radiograph; (2) suspected airway and lung malformations; (3) serious complications associated with pneumonia occurred; (4) routine treatment was ineffective after excluding other diseases such as interstitial lung disease, pulmonary tuberculosis, so on. In our study, all these RMPP patients underwent chest CT scan before FOB therapy [15, 16]. PB was diagnosed based on as follows: (1) manifestations of bronchoscopy: respiratory mucosa congestion, edema and increased mucus discharge; the bronchial lumen was blocked by inflammatory BCs, which were removed by biopsy forceps and expanded to “branch-like” plastic after immersing in 9 g/L saline; (2) Histopathology: the inflammatory BCs was composed of extensive inflammatory cells (predominantly eosinophils and neutrophils) and exfoliated epithelial cells, the immunohistochemistry was CD3(+), CD20(+), CD68(+), MPO(+).

The criteria for the use of glucocorticoids including [17]: (1) wheezing with increased respiratory secretions; (2) severe pneumonia with obvious toxic symptoms, such as hypoxic toxic encephalopathy, shock sepsis, acute respiratory distress syndrome (ARDS); (3) a large amount of exudation in the chest for a short time. (4) persistent high fever due to a strong inflammatory reaction. The criteria for the use of intravenous gamma globulin (IVIG) [18, 19]: (1) MPP complicated with central nervous system, autoimmune hemolytic anemia, immune thrombocytopenic purpura, et al. (2) IVIG should be used as an adjuvant for the treatment of SMPP or RMPP in children with extrapulmonary lesions.

### Potential predictive variables

All the patients’ medical data were collected at hospital admission, including demographic information, clinical symptoms and signs, laboratory findings, imaging features and managements. Potential predictive variables included the following 26 characteristics: sex, age, peak body temperature, presence of hypoxemia, white blood cell (WBC), neutrophil ratio (N%), lymphocyte ratio (L%), platelet counts (PLT), erythrocyte sedimentation rate (ESR), C-reactive protein (CRP), procalcitonin (PCT), interleukin-6 (IL-6), lactic acid (La), aspartate aminotransferase (AST), alanine aminotransferase (ALT), creatine kinase (CK), Creatine kinase isomer-MB (CK-MB), lactic dehydrogenase (LDH), ferritin (FER), activated partial thromboplastin time (APTT), fibrinogen (FG), D-dimer, Immunoglobulin E (IgE) and 3 abnormal indicators on chest computed tomography (CT), including atelectasis, pleural thickening and pleural effusion.

### Variable selection and model construction

All the 374 patients in the development dataset were analyzed for variable selection and risk prediction development. Least Absolute Shrinkage and Selection Operator (LASSO) regression was applied to select the optimal prediction factors of PB from 26 features. LASSO is a logistic regression model which can select predictors by penalized the coefficients of all the variables. With larger penalties, the coefficients of weaker factors shrink toward zero and finally the variables remained in the model were selected. Cross-validation was used to determine the appropriate adjustment parameter (λ) for LASSO logistic regression [20]. Variables identified by LASSO regression analysis were applied to establish logistic regression model and presented with a nomogram. Furthermore, these selected continuous predictors (i.e., peak body temperature, N%, PLT, IL-6 and LDH) were categorized into three groups based on tertile after being assessed by restricted cubic splines to evaluate the linear relationship assumptions [21, 22]. Additionally, the degree of multicollinearity among variables was evaluated by the variance inflation factor (VIF) in the multivariable logistic regression analysis. If VIF was > 10, then multicollinearity was high [23].

### Assessment of accuracy

The performance of the prediction model was evaluated from discrimination ability, calibration ability and clinical value. The discrimination ability was evaluated through receiver operator characteristic (ROC) analysis with area under the curve (AUC) and the calibration plot accompanied with the Hosmer–Lemeshow test was applied to assess the calibration ability. The model was validated using bootstrap method with 1000 resamples to quantify any overfitting [24]. Furthermore, ROC curves analysis were performed to compare the discrimination ability between the nomogram, clinical features and laboratory biomarkers with area under curve (AUC) value. A decision curve analysis (DCA) was applied to evaluate the clinical utility of the nomogram based on its net benefits at different threshold probabilities and was also compared between the nomogram, clinical features and laboratory biomarkers.

### Validation of the nomogram

To validate the accuracy of the nomogram, we collected 173 patients’ medical records from our hospital that were not included in the development dataset. Data for the 173 RMPP patients were analyzed as the validation dataset from January 2020 to June 2021. And the criteria for the cases in the validation dataset was same as the development dataset. The variables required for evaluating the prediction from the validation dataset were collected and calculated as described herein for the development dataset.

### Statistical analysis

Continuous variables were expressed as mean ± standard deviation (SD) or median values (interquartile range) and assessed by independent group *t* tests or Mann–Whitney U test. Categorical variables were expressed as percentage (%) and assessed by Chi-squared tests or Fisher’s exact test. Statistical analysis was carried out using SPSS 26.0 and R software (version 4.0.5, http://www.r-project.org) was used to perform all the graphics based on R packages “foreign”, “rms”, “ggplot2”, “pROC”, “car” and “glmnet”. Program on R package was  provided as Additional file 1. A two-sided α less than 0.05 were considered as statistically significant.

## Results

### Patient characteristics

The clinical characteristics and laboratory findings of the development dataset are summarized in Tables 1 and 2. The development dataset included 374 patients (237 in the RMPP group, 137 in the RMPP combined with PB group) with a mean age of 6.6 years and 185(49.5%) were men. The mean duration of fever and hospitalization were 9.8 ± 2.5 and 8.2 ± 2.7 days, respectively. Among these 374 patients, 82 (21.9%) patients underwent multiple FOB and BAL procedure and 52 (13.1%) patients suffered from hypoxemia. The incidence of pulmonary atelectasis, pleural effusion and thickening were 29.4%, 27.8% and 66.8% respectively. There was no significant difference in age, sex, incidence of fever and cough between the two groups (*P* > 0.05). The peak body temperature, duration of fever and hospitalization, incidence of hypoxemia, pulmonary atelectasis and pleural effusion, administration of glucocorticoid and intravenous immunoglobulin (IVIG) were significantly higher in the RMPP combined with PB group than that in the RMPP group. Compared with the RMPP group, the RMPP combined with PB group showed higher levels of N% (72.0 vs 67.0%), CRP (32.8 vs 22.6 mg/L), PCT (0.30 vs 0.12 ng/mL), IL-6 (44.0 vs 22.5 pg/ml), AST (39.0 vs 30.0 U/L), ALT (16.0 vs 14.0 U/L), CK (122.0 vs 94.0 U/L), LDH (483.0 vs 392.0 U/L), D-dimer (0.2 vs 0.1 mg/L), lower levels of L% (20.0 vs 24.3%) and PLT count (249.0 vs 279.0 × 10^9^/L).

**Table 1:** Clinical characteristics and imaging features of patients between RMPP combined with PB group and RMPP group in the development dataset

Clinical characteristics	Patients (n = 374)	RMPP combined with PB (n = 137)	RMPP (n = 237)	*P*
Age, years	6.6 ± 2.7	6.8 ± 2.6	6.5 ± 2.8	0.307
Sex (male/female)	185/189	64/73	121/116	0.419
Fever (n, %)	374 (100%)	137 (100%)	237 (100%)	1.000
Cough (n, %)	374 (100%)	137 (100%)	237 (100%)	1.000
Patients with multiple FOB (n, %)	82 (21.9%)	59 (43.1%)	23 (9.7%)	0.000
Peak body temperature (°C)	39.8 ± 0.7	40.1 ± 0.6	39.7 ± 0.6	0.000
Duration of fever (days)	9.8 ± 2.5	10.6 ± 2.9	9.3 ± 2.1	0.000
Duration of hospitalization (days)	8.2 ± 2.7	9.3 ± 3.0	7.5 ± 2.2	0.000
Fever duration before FOB (days)	8.0 ± 2.4	8.2 ± 2.6	7.9 ± 2.3	0.391
Hypoxemia (n, %)	52 (13.1%)	25 (18.2%)	24 (10.1%)	0.025
Glucocorticoid (n, %)	265 (70.9%)	130 (94.9%)	135 (56.9%)	0.000
IVIG (n,%)	28 (7.5%)	20 (14.6%)	8 (3.4%)	0.000
Admission to ICU (n, %)	14 (3.7%)	6 (4.4%)	8 (3.4%)	0.622
Atelectasis (n, %)	110 (29.4%)	64 (46.7%)	46 (19.4%)	0.000
Pleural effusion (n, %)	104 (27.8%)	54 (39.4%)	50 (21.1%)	0.000
Pleural thickening (n, %)	250 (66.8%)	91 (66.4%)	159 (67.1%)	0.851

Data are presented as mean ± SD and *n* (%). Differences between groups were determined by the independent group t tests(mean ± SD) and Chi-squared tests (proportions). FOB Fiberoptic Bronchoscopy, IVIG intravenous immunoglobulin, ICU intensive care unit

**Table 2:** Laboratory findings of patients between RMPP combined with PB group and RMPP group in the development dataset

Laboratory information	Patients (n = 374)	RMPP combined with PB (n = 137)	RMPP (n = 237)	*P*
WBC (× 10^9^/L)	7.4 (5.9–9.4)	7.4 (5.8–9.3)	7.4 (5.9–9.5)	0.661
N%	68.6 (60.0–76.0)	72.0 (65.0–78.4)	67.0 (57.1–73.1)	0.000
L%	22.0 (16.8–29.3)	20.0 (15.0–25.0)	24.3 (17.4–31.6)	0.000
PLT (× 10^9^/L)	267.0 (221.0–335.3)	249.0 (205.0–311.0)	279.0 (232.0–356.5)	0.000
ESR (mm/h)	29.5 (22.0–40.0)	30.0 (22.0–41.0)	29.0 (23.0–40.0)	0.841
CRP (mg/L)	25.9 (11.7–46.0)	32.8 (18.0–58.8)	22.6 (9.7–39.4)	0.000
PCT (ng/mL)	0.16 (0.10–0.33)	0.30 (0.10–0.60)	0.12 (0.07–0.24)	0.000
IL-6 (pg/mL)	28.6 (16.4–52.3)	44.0 (27.3–78.9)	22.5 (14.5–38.4)	0.000
La (mol/L)	2.6 (2.1–3.1)	2.5 (2.1–3.1)	2.6 (2.1–3.1)	0.459
AST (U/L)	31.0 (25.0–44.0)	39.0 (29.0–59.0)	30.0 (24.0–39.0)	0.000
ALT (U/L)	15.0 (12.0–24.0)	16.0 (12.0–28.5)	14.0 (11.0–20.0)	0.007
CK (U/L)	99.0 (67.0–184.3)	122.0 (73.5–245.5)	94.0 (66.0–147.5)	0.003
CKMB (U/L)	4.0 (3.7–4.0)	4.0 (3.0–4.0)	4.0 (4.0–4.0)	0.335
LDH (U/L)	427.0 (333.8–560.5)	483.0 (375.0–641.5)	392.0 (313.5–513.0)	0.000
FER (ng/L)	132.7 (89.6–227.4)	142.0 (74.2–261.3)	132.2 (98.3–196.5)	0.639
APTT (S)	29.5 (26.2–32.9)	30.1 (26.1–33.9)	29.1 (26.4–32.6)	0.262
FG (g/L)	4.1 (3.6–4.6)	4.2 (3.7–4.7)	4.0 (3.7–4.5)	0.161
D-dimer (mg/L)	0.2 (0.1–0.4)	0.2 (0.1–0.6)	0.1 (0.1–0.3)	0.000
IgE (IU/L)	94.7 (39.7–242.3)	108.0 (38.9–260.9)	89.8 (40.2–227.7)	0.586

Data are presented as median (25th–75th percentile). Differences between groups were determined by the Mann–Whitney U test (medians).WBC White blood cell, N Peripheral neutrophils, L Peripheral lymphocytes, PLT Platelets, ESR Erythrocyte sedimentation rate, CRP C-reactive protein, PCT Procalcitonin, IL-6 Interleukin (IL)-6, La Lactic acid, AST Aspartate aminotransferase, ALT Alanine aminotransferase, CK creatine kinase, CKMB Creatine kinase isomer-MB, LDH Lactic dehydrogenase, FER Ferritin, APTT activated partial thromboplastin time, FG Fibrinogen, IgE Immunoglobulin E

### Predictor selection and construction of nomogram

**Fig. 2:**
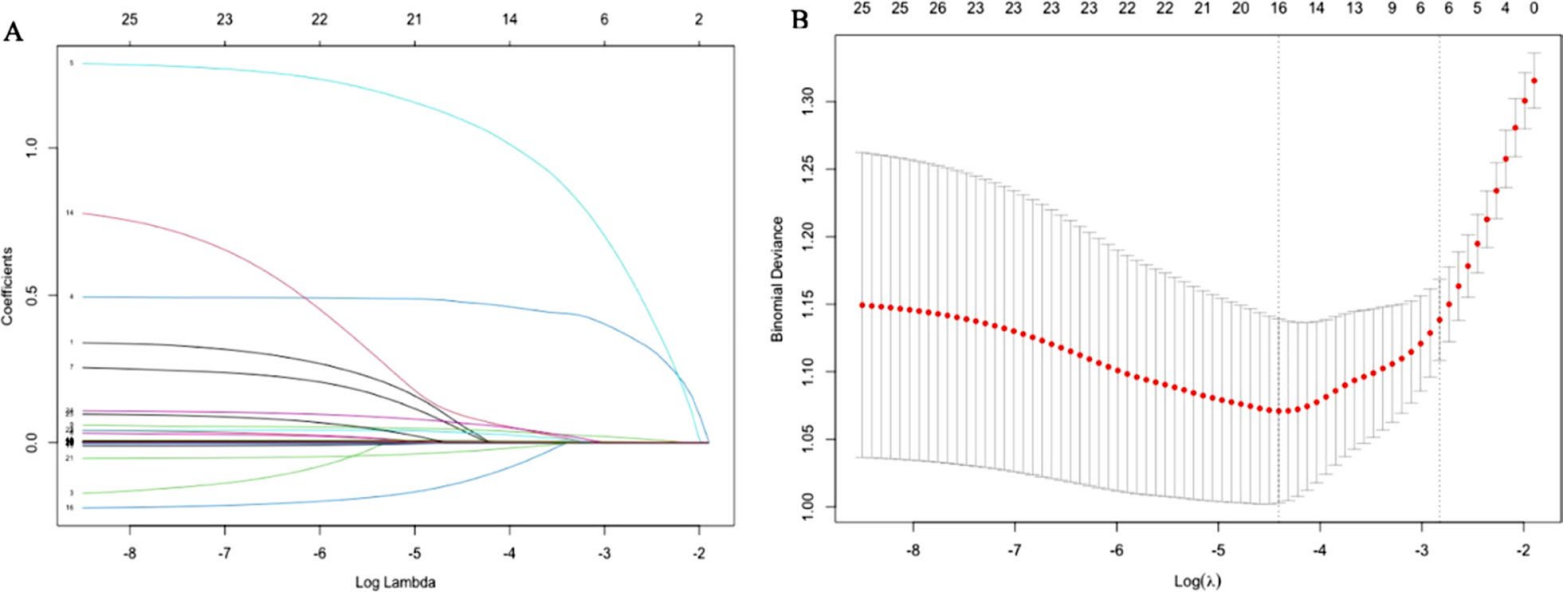
Variable selection using least absolute shrinkage and selection operator (LASSO) logistic regression. **A** LASSO coefficient profiles of the 26 variables. With larger penalties, the coefficients of an increasing number of variable are compressed; finally, most of the variable coefficients are compressed to zero. **B** The best penalty coefficient lambda was selected using a tenfold cross-validation and minimization criterion. By verifying the optimal parameter (lambda) in the LASSO model, the binomial deviance curve was plotted versus log(lambda) and dotted vertical lines were drawn based on 1 standard error criteria. 6 variables with nonzero coefficients were selected by optimal lambda

Twenty-six variables, including sex, age, peak body temperature, presence of hypoxemia, WBC, N%, L%, PLT, ESR, CRP, PCT, IL-6, La, AST, ALT, CK, CK-MB, LDH, FER, APTT, FG, D-dimer, IgE, atelectasis, pleural thickening and pleural effusion (Fig. 2-A), which might predict PB formation were included in the LASSO regression based on the development dataset. With larger penalties, LASSO compresses the coefficients of most variables to 0 and eventually variables with non-zero coefficients were screened. Cross-validation was used to determine the appropriate adjustment parameter (λ) for LASSO regression and finally six variables were selected as significant predictors of PB formation (Fig. 2-B). Continuous predictors including peak body temperature, N%, PLT, IL-6 and LDH were categorized into three groups based on tertile after being assessed using restricted cubic splines to evaluate the linear relationship assumptions. In terms of the collinearity diagnosis, the VIFs of the six predictors varied between 1.56 and 9.08, confirming that there was no collinearity.

**Fig. 3:**
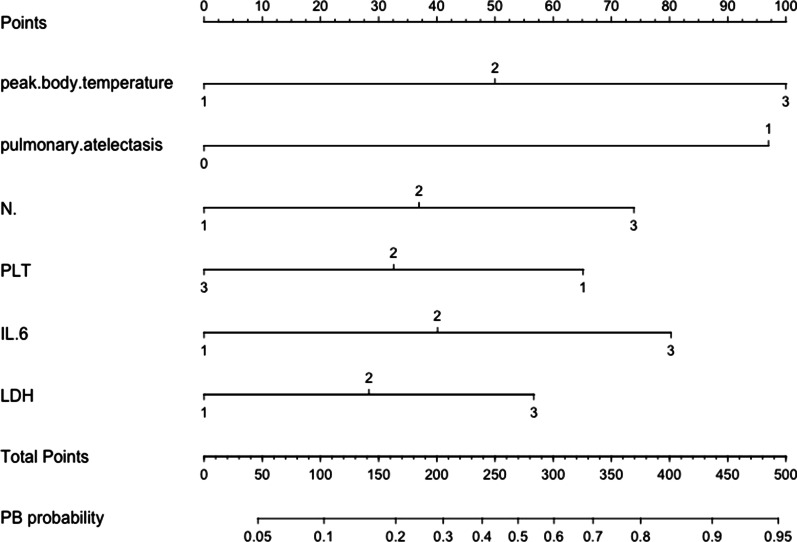
Nomogram to predict PB among RMPP children was constructed based on 6 independent predictors. Mark the value of these included factors on the corresponding axis. Draw a vertical line from the value to the top lines and get corresponding points. Then, sum the points from each variable value. Locate the sum on the total points scale and project it vertically on the bottom axis to obtain a PB risk

Then the six selected variables were applied to establish logistic regression model and presented with a nomogram (Fig. 3). These six variables included peak body temperature, N%, PLT, IL-6, LDH and pulmonary atelectasis (Table 3). The final predictive model incorporating the six factors was shown as a nomogram in Fig. 3. Total points based on the sum of the points for each predictor in this nomogram were associated with the risk of PB.

**Table 3:** Multivariable Logistic Regression Model for Predicting PB in 374 Patients with RMPP

Variables	OR (95% CI)	*P* value
Peak body temperature (°C)		0.007
Tertile 1 (lowest to 39.5)	1	
Tertile 2 (39.5 to 40.0)	2.045 (0.982–4.259)	0.056
Tertile 3 (40.0 to highest)	3.690 (1.617–8.416)	0.002
N%		0.029
Tertile1 (lowest to 64.0)	1	
Tertile 2 (64.0 to 73.0)	1.574 (0.839–2.952)	0.158
Tertile 3 (73.0 to highest)	2.459 (1.268–4.769)	0.008
IL-6 (pg/mL)		0.020
Tertile 1 (lowest to 21.0)	1	
Tertile 2 (21.0 to 42.4)	1.667 (0.854–3.255)	0.134
Tertile 3 (42.4 to highest)	2.579 (1.323–5.029)	0.005
LDH (U/L)		0.006
Tertile 1 (lowest to 361.0)	1	
Tertile 2 (361.0 to 508.0)	1.910 (0.987–3.696)	0.055
Tertile 3 (508.0 to highest)	3.000 (1.535–5.861)	0.001
PLT(× 10^9^/L)		0.029
Tertile 1 (lowest to 235.0)	1	
Tertile 2 (235.0–306.0)	0.544 (0.298–0.994)	0.048
Tertile 3 (306.0 to highest)	0.433 (0.225–0.832)	0.012
Atelectasis (n, %)	3.836 (2.241–6.566)	< 0.001

*OR* odds ratio, *CI* confidence interval

### Performance of the nomogram

**Fig. 4:**
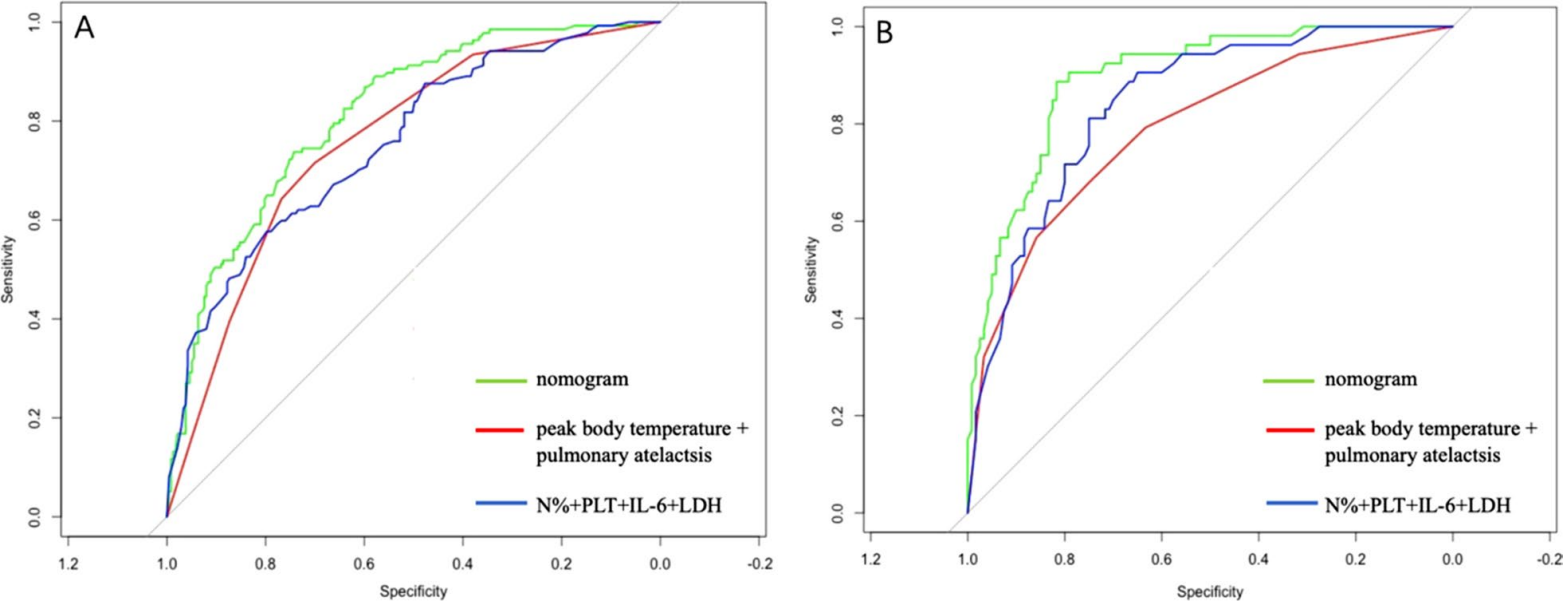
The ROC curves of the nomogram from the development cohort (**A**) and the validation cohort (**B**). ROC: receiver operating characteristics

**Fig. 5:**
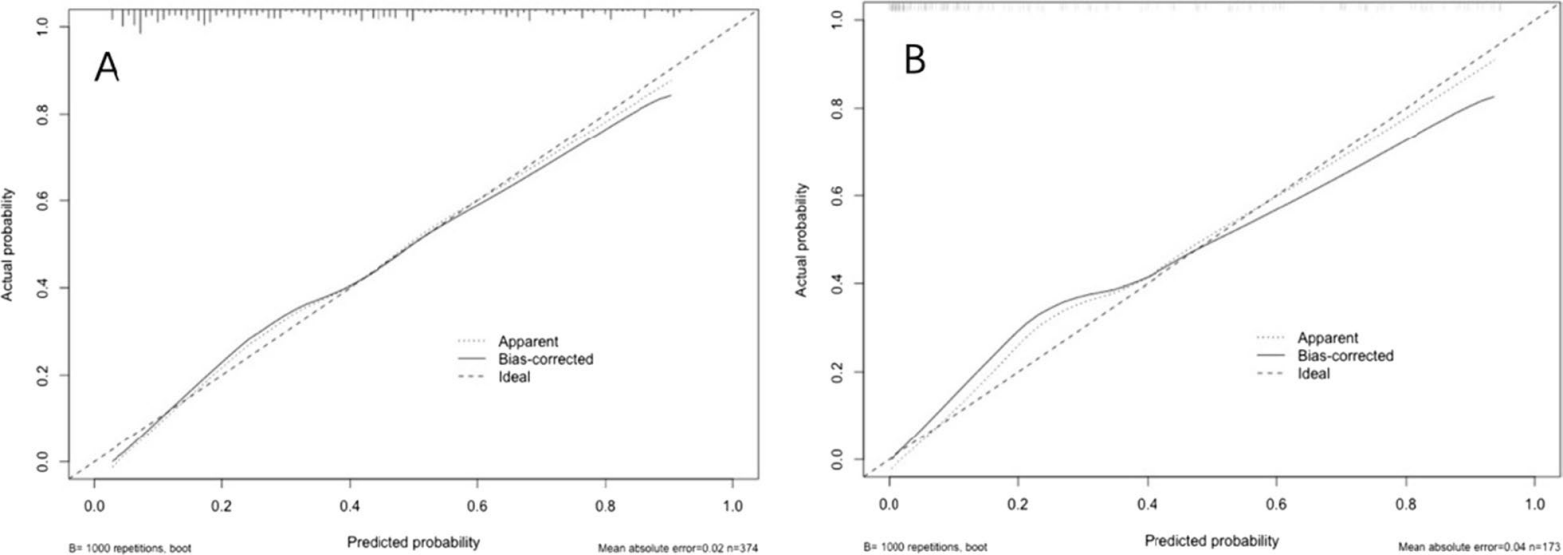
Calibration plot of PB risk nomogram in the development cohort (**A**) and validation cohort (**B**). The ideal outcome (dashed line), the observed outcome (fine dashed line), and the bias-corrected outcome (solid line) are depicted

By internal bootstrap validation with 1000 resamples, the mean AUC of the nomogram based on the development dataset was 0.813 (95% CI 0.769–0.856) (Fig. 4-A), with good discrimination ability for predicting PB in RMPP patients. Furthermore, the calibration plot (Fig. 5-A) and Hosmer‐Lemeshow test (*P* = 0.217) of the prediction model showed good consistency between the predicted probability and actual probability.

The validation dataset included 173 patients, of which 120 cases were in the RMPP group and 53 cases in the RMPP combined with PB group. The average age of the validation dataset was 6.7 years and 86 (49.7%) were men. The clinical and laboratory characteristics of the dataset was shown in Tables 4 and 5. The accuracy of the nomogram in the validation dataset was similar to that of the development dataset with an AUC value of 0.895 (95% CI 0.847–0.943) (Fig. 4-B). To evaluate the discrimination ability of nomogram, ROC analysis was compared between nomogram and other model both in the development dataset and validation dataset. ROC analysis indicated that the nomogram showed better discrimination ability compared with model of peak body temperature + pulmonary atelactsis and another model of N% + PLT + IL-6 + LDH in development dataset (AUC 0.813 vs 0.757 vs 0.754) and validation dataset (AUC 0.895 vs 0.789 vs 0.842) (Table 6, Fig. 4).

**Table 4:** Clinical characteristics and imaging features of patients between RMPP combined with PB group and RMPP group in the validation dataset

Clinical characteristics	Patients (n = 173)	RMPP combined with PB (n = 53)	RMPP (n = 120)	*P*
Age, years	6.7 ± 2.6	6.9 ± 2.5	6.5 ± 2.6	0.269
Sex (male/female)	86/87	25/28	61/59	0.657
Fever (n, %)	173 (100%)	53 (100%)	120 (100%)	1.000
Cough (n, %)	173 (100%)	53 (100%)	120 (100%)	1.000
Patients with multiple FOB (n, %)	49 (28.3%)	24	23 (19.2%)	0.000
Peak body temperature, °C	39.8 ± 0.5	40.1 ± 0.6	39.6 ± 0.4	0.000
Duration of fever, days	10.0 ± 2.4	11.3 ± 2.7	9.4 ± 2.0	0.000
Duration of hospitalization, days	7.8 ± 2.2	8.8 ± 2.6	7.4 ± 1.9	0.000
Fever duration before FOB, days	8.0 ± 2.5	8.3 ± 3.2	7.9 ± 2.0	0.398
Hypoxemia (n, %)	25 (14.5%)	11 (20.8%)	14 (11.7%)	0.117
Glucocorticoid (n, %)	134 (77.9%)	49 (92.5%)	85 (71.4%)	0.002
IVIG (n, %)	9 (5.2%)	5 (9.4%)	4 (3.3%)	0.135
Admission to ICU (n, %)	5 (2.9%)	2 (3.8%)	3 (2.5%)	0.643
Atelectasis (n, %)	67 (39.0%)	36 (67.9%)	31 (26.1%)	0.000
Pleural effusion (n, %)	59 (34.3%)	32 (60.4%)	27 (22.7%)	0.000
Pleural thickening (n, %)	101 (58.7%)	30 (56.6%)	71 (59.7%)	0.707

Data are presented as mean ± SD and n (%). Differences between groups were determined by the independent group t tests(mean ± SD) and Chi-squared tests (proportions). FOB Fiberoptic Bronchoscopy, IVIG intravenous immunoglobulin, ICU intensive care unit

**Table 5:** Laboratory findings of patients between RMPP combined with PB group and RMPP group in the validation dataset

Laboratory information	Patients (n = 173)	RMPP combined with PB (n = 53)	RMPP (n = 120)	*P*
WBC (× 10^9^/L)	7.1 (5.6–9.6)	7.5 (5.8–9.3)	7.5 (5.9–9.5)	0.552
N%	66.3 (57.5–74.2)	70.5 (65.0–78.2)	66.7 (56.7–72.9)	0.000
L%	23.9 (17.1–32.2)	20.0 (15.0–25.0)	24.4 (17.5–32.4)	0.003
PLT (× 10^9^/L)	271.0 (221.0–343.0)	249.5 (205.8–310.0)	280.0 (232.0–358.8)	0.005
ESR (mm/h)	27.0 (21.0–37.0)	29.0 (22.0–41.0)	29.0 (22.0–40.0)	0.567
CRP (mg/L)	25.4 (15.7–46.6)	32.5 (18.0–58.6)	22.5 (8.9–38.9)	0.000
PCT (ng/mL)	0.14 (0.08–0.30)	0.30 (0.10–0.60)	0.12 (0.07–0.24)	0.000
IL-6 (pg/mL)	25.0 (14.5–48.4)	43.5 (27.1–78.2)	22.2 (14.1–38.4)	0.000
La (mol/L)	2.6 (2.1–3.3)	2.5 (2.1–3.1)	2.6 (2.1–3.2)	0.157
AST (U/L)	32.0 (25.0–41.0	39.0 (29.0–59.0)	30.0 (23.0–39.0)	0.000
ALT (U/L)	14.0 (10.0–27.0)	16.0 (12.0–28.3)	14.0 (11.0–20.0)	0.000
CK (U/L)	103.0 (58.0–197.5)	126.0 (74.8–244.3)	91.0 (64.0–144.5)	0.001
CKMB (U/L)	4.0 (3.0–5.0)	4.0 (3.0–4.0)	4.0 (4.0–4.0)	0.106
LDH (U/L)	404.0 (323.0–551.0)	482.0 (375.5–630.3)	389.0 (310.5–505.5)	0.000
FER (ng/L)	151.1 (106.4–252.6)	131.8 (74.3–254.7)	130.2 (95.0–195.4)	0.326
APTT (S)	30.6 (26.7–34.8)	30.1 (26.1–34.1)	29.2 (26.4–32.6)	0.090
FG (g/L)	4.0 (3.4–4.4)	4.2 (3.7–4.6)	4.0 (3.6–4.5)	0.064
D-dimer (mg/L)	0.2 (0.1–0.5)	0.2 (0.1–0.6)	0.1 (0.1–0.3)	0.000
IgE (IU/L)	122.3 (48.2–285.0)	108.0 (39.0–252.3)	89.8 (40.2–221.6)	0.630

Data are presented as median (25th–75th percentile). Differences between groups were determined by the Mann–Whitney U test (medians).WBC White blood cell, N Peripheral neutrophils, L Peripheral lymphocytes, PLT Platelets, ESR Erythrocyte sedimentation rate, CRP C-reactive protein, PCT Procalcitonin, IL-6 Interleukin (IL)-6, La Lactic acid, AST Aspartate aminotransferase, ALT Alanine aminotransferase,CK creatine kinase, CKMB Creatine kinase isomer-MB, LDH Lactic dehydrogenase, FER Ferritin, APTT activated partial thromboplastin time, FG Fibrinogen, IgE Immunoglobulin E. Data are presented as mean ± SD

**Table 6:** Assessing the prediction performance of the the nomogram and other model in training cohort and validation cohort

Dataset	Model	Odds ratios (95% CI)
Development dataset	Nomogram	0.813 (0.769–0.856)
	Model 2	0.757 (0.709–0.806)
	Model 3	0.754 (0.704–0.815)
Validation dataset	Nomogram	0.895 (0.847–0.943)
	Model 2	0.789 (0.706–0.863)
	Model 3	0.848 (0.783–0.902)

Mode 2: peak body temperature + pulmonary atelectasis; Mode 3: N% + PLT + IL-6 + LDH

**Fig. 6:**
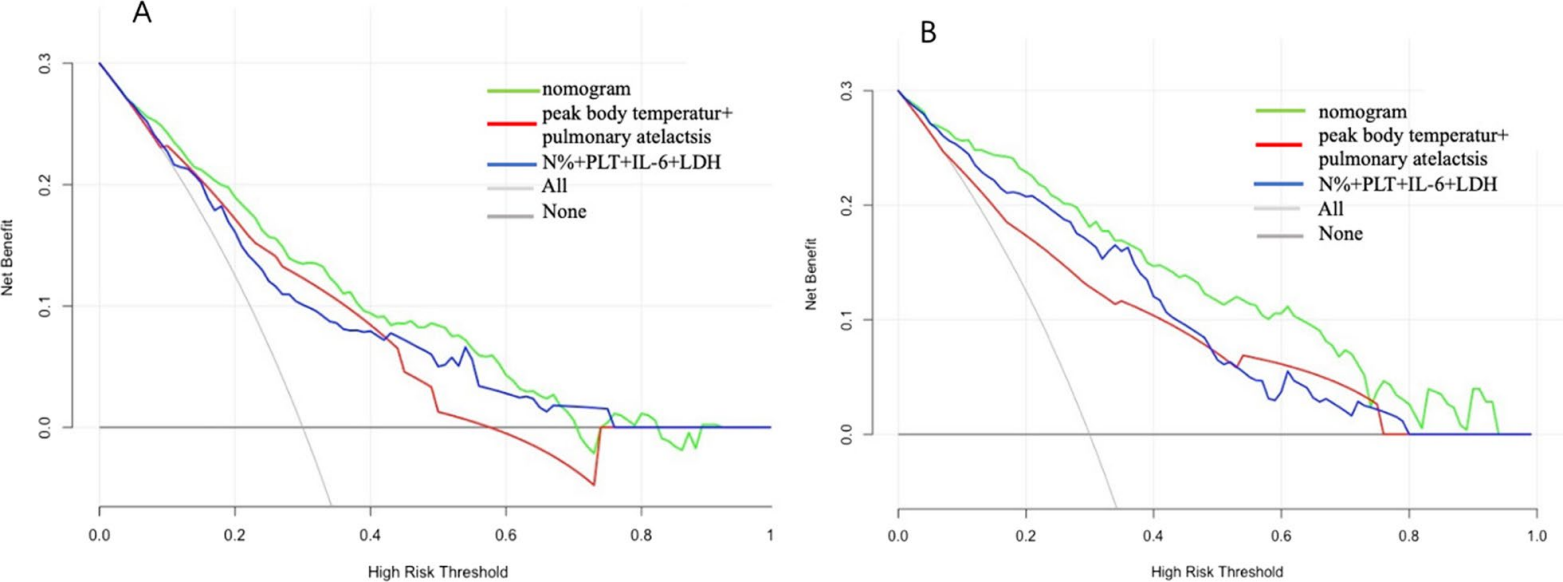
Decision curve analysis for the PB risk nomogram. The y-axis measured the net benefit. The black solid line represented the assumption that all patients had no PB. The gray solid line represented the assumption that all patients had PB. **A** From the development cohort and **B** from the validation cohort

The calibration plot (Fig. 5-B) accompanied with the Hosmer‐Lemeshow test (*P* = 0.183) showed that the prediction model fits well in the validation dataset. We applied DCA curve to evaluate the clinical value of the prediction model. The DCA curve showed obvious net benefits of the predictive nomogram and were significantly higher than those of the two extreme cases. DCA was also applied to compare the clinical usefulness of the nomogram to that of other models (Fig. 6).

## Discussion

In this study, we developed and validated a nomogram to predict the development of PB in RMPP children. Twenty-six potential variables were included in LASSO regression, finally six variables were determined to construct the nomogram, including peak body temperature, N%, PLT, IL-6, LDH and pulmonary atelectasis. The performance of this nomogram was satisfactory with good accuracy and discrimination both in the development and validation dataset. The six variables required for the construction of the nomogram were easily available at admission, suggesting that it could be a convenient tool to help physicians estimate the risk of PB in RMPP children.

Previously, PB characterized by formation of BCs in the airway was usually reported in children with surgically palliated congenital heart disease, especially those after the Fontan procedure [25]. The clinical manifestations of PB are diverse, including fever, cough, dyspnea or respiratory distress among which rapid progression to hypoxemia can be applied as a strong indicator of PB. In recent years, with the development of FOB therapy and the rising incidence of RMPP, BCs formation is commonly seen in RMPP patients [26–30]. RMPP children with BCs formation usually presented with glucocorticoids-resistant, excessive inflammation, delayed clinical and radiographic resolution and long-standing pulmonary sequelae [31]. In our study, 52 (13.1%) cases suffered from hypoxemia and Li W et al. [30] revealed that in their study all the 15 children with PB showed no signs of hypoxemia, among which MP infection accounted for 86.7% (13/15). The incidence of hypoxemia and critically ill was lower than previous description (58.3%, 14/24 cases) [32]. The possible explanation may be attributed to the following two aspects. On one hand, the clinical manifestation of PB depends on the location and degree of bronchial obstruction, ranging from fragmented partial BCs to a large and complete cast that fills the entire airway. Previous studies [33] found that in PB caused by different pathogens, MP infection prone to cause fragmented partial BCs, which may be characteristic of MP infection related PB, and thus symptoms such as acute respiratory distress are less likely to occur. On the other hand, with the better understanding and application of FOB, the studies on PB caused by respiratory infection gradually increased, and clinicians' understanding of the development of PB in severe pneumonia also improved. Then rapid FOB treatment contributed to early effective intervention and prevented the development of respiratory failure.

Although the underlying mechanisms of BCs formation in patients with MP infection are still uncertain, previous studies have shown that MP infection not only directly cause necrosis of the airway epithelial, but also induce cilia removal dysfunction to promote the formation of mucus plug by the excessive inflammation [34, 35]. Previous studies have reported that some clinical variables and biomarkers related to inflammation response were associated with mucus plug formation or PB. The variables that have been investigated included age, fever duration, presence of complications, L%, CRP, LDH, prealbumin, IL-10 and IFN-γ [29, 36, 37]. In this study, we found that RMPP patients who had higher peak body temperature, N%, IL-6, LDH, presence of atelectasis and lower level of PLT count are at higher risk of developing PB. These findings suggest that the excessive inflammation play an important role in the formation of BCs.

An increase in body temperature can increase patient’s basal metabolic rate, enhance the excitability of central nervous system and reduce body defense function. Patients with persistent high fever are prone to experience irritability, convulsions, tachycardia, tachypnea, dehydration and even life-threatening complications. MP can act as a pyrogen in vitro, causing body fever and stimulating the body to produce a large number of inflammatory factors. Currently, persistent high fever is generally considered to be related to the excessive inflammatory response caused by MP. Recent studies have identified that compared with general MPP (GMPP) patients, fever for more than 10 days and high fever were risk factor for RMPP [8, 28, 38]. Xu et al. [29] found that RMPP patients with mucus plug had higher peak body temperature and our results were similar to this. In our research, the RMPP combined with PB group exhibited a significant higher peak body temperature than that in the RMPP group (40.1 vs 39.6 °C). Moreover, the nomogram constructed based on peak body temperature showed good ability both in accuracy and discrimination in the present study. Peak body temperature act as a risk factor for BCs formation, may be because that the essence of BCs is the accumulation of thick mucus plugs. The loss of water from the respiratory tract resulted from high fever and inadequate influid intake can lead to thickness of the mucus secretions and thus promote the formation of BCs.

Blood cell analysis is a most common and easily accessible test to assist the diagnosis of infectious disease. Previous studies have found that higher neutrophil was positively correlated with excessive inflammation and disease severity in children with MPP [8, 28, 39]. At present, most studies have compared the proportion of neutrophils in children with RMPP and in children with RMPP accompanied by mucus plug, and found that the neutrophils in the latter group were significantly increased [29, 36]. Zhang et al. [33] identified that percentage of neutrophils (N > 70%) was independent risk factors for PB caused by MP infection which was consistent with our results. The reason may be that increased neutrophils in the acute stage can injure the airways through the release of proteases, reactive oxygen and inflammatory cytokines [40]. Interleukin is one kind of cytokines and exert an important role in the immunopathogenesis of MP infection [41, 42]. IL-6, as a member of the interleukin family, was observed elevated in MPP patients with excessive inflammation [8, 43, 44]. Ling et al. [44] showed that in children with MPP, the cutoff value of IL-6 for MPP with hypoxia was 25.47 pg/ml and in Zhang et al.’s [29] study, IL-6 was identified as a predictor for RMPP with a cutoff value of 14.75 pg/ml. In our research, IL-6 was observed significantly elevated in the PB group and determined as a predictor for PB. The increase of N% and IL-6 reflect the excessive inflammatory response which can promote the formation of BCs. LDH, a nonspecific inflammatory biomarker, exists within the cytoplasm and is generally considered as a reliable indicator to assess disease severity as it can be released to the serum after cell damage [3, 45]. Various studies have identified higher level of LDH as a risk factor for RMPP [5, 8, 9, 38]. Recently, it has been reported that higher LDH level was an independent risk factor for MPP mucus plug formation [29, 36] and our results were consistent with these researches. These elevated inflammation biomarkers indicated an excessive inflammation response, leading to serious airway damage and ciliary clearance dysfunction, eventually resulting in the formation of BCs.

Interestingly, we found that the PLT count in the RMPP combined with PB group was significantly lower than that in the RMPP group, and lower PLT was considered to be a risk factor for predicting PB. Similar results were also reported in previous research [29, 36, 46, 47]. Xu et al. and Hua [29, 47] found that in children with RMPP, the platelet count was significantly lower in the mucus plug group or PB group than that in the non-mucus plug group or non-PB group(249 vs 288 × 10^9^/L and 244.5 vs 288.4 × 10^9^/L). Yang et al. [46] compared the PLT level among severe MPP (SMPP) group, MPP group and control group both in the acute stage and the recovery stage. In the acute period, compared with the control group, the SMPP group had a lower PLT level but the MPP group had a higher PLT level. In the recovery stage, the PLT level in the SMPP group significantly increased and was the highest among the three groups. It seems that in MP-infected patients, PLT counts may be related to the severity of inflammation and the stage of disease. We speculated that the reasons of PLT count decline in the acute phase might be related to the following three aspects. On one hand, the hyperactivity of humoral immune function caused by MP infection causes B-lymphocytes to produce platelet-related antibodies, thus inducing the formation of antigen–antibody complexes. These antigen–antibody complexes can be absorbed by macrophages via Fc receptors, then be gobbled up and destroyed in the spleen. On the other hand, Large amounts of oxygen free radicals produced by local hypoxia and carbon dioxide retention due to the formation of BCs and mucus plug, can attack PLT and increase its damage. In addition, the formation of mucus plug and release of a variety of coagulation active substances caused by hypoxia in SMPP patients might lead to excessive platelet depletion. Therefore, more attention should be paid to RMPP patients with a lower PLT level in the acute stage, as it may be a predictor for developing PB.

The imaging features of children with MPP are diverse and nonspecific, including bronchopneumonia, interstitial lung lesions, and segmental or lobular infiltration. Patients with RMPP often present with more severe pulmonary complications. Previous studies reported that the incidence of serious radiographic findings, including pleural effusion, lobar atelectasis, consolidation and pleural thickening in the RMPP group were significantly higher than that in the GMPP group [8, 38]. Xu et al. [29] demonstrated that complications including pulmonary atelactesis and pleural effusion were the independent risk factors for developing bronchus mucus plug in children with RMPP and atelactesis had the top score and the highest weight in their prediction nomogram. In our research, the incidence of pulmonary atelectasis, pleural effusion and thickening were 30.3%, 27.6% and 65.8% respectively and atelectasis was a predictor for PB. Airway injury and ciliary dysfunction play an important role in the formation of BCs and accumulation of necrosis or inflammatory substances is also the pathogenesis of pulmonary atelectasis. Therefore, it’s very likely that BCs have formed when patients with RMPP shows atelectasis in chest imaging.

FOB surgery has shown significant efficacy in the treatment of PB, including direct clearance of BCs to improve pulmonary ventilation and clearance of various inflammatory factors, contributing to faster recovery of clinical manifestations and shorter hospital stay. However, FOB is an invasive procedure, the indications and risk–benefit ratio should be considered carefully. The purpose of this nomogram based on clinical and laboratory characteristics is to help clinicians identify potential PB patients for appropriate management, including inflammatory modulators and FOB therapy. If the patient’s estimated risk for PB formation is low, the clinicians may choose to monitor, whereas high-risk estimates might support aggressive treatment and FOB investigation.

Although our nomogram performed well in both the development and validation dataset, there are several limitations to this study. Firstly, the data for the nomogram development and validation are from one medical center, which could limit the generalizability of it in other areas. Secondly, the nomogram is based on a retrospective study and individuals with incomplete data are excluded, which may lead to selection bias. Thirdly, some patients may be coinfected with other pathogens, which could not be detected precisely. Multicenter and prospective validation studies of the nomogram should be completed in the future.

## Conclusions

In this study, we established and validated a nomogram for predicting PB formation among patients with RMPP. The nomogram was constructed based on 6 variables commonly measured on admission to the hospital, including peak body temperature, N%, PLT, IL-6, LDH and pulmonary atelectasis. It performs well on discrimination ability, calibration ability and clinical value and dose have the potential for the early identification of PB, thereby making contribution to timely intervention and appropriate treatment.

## Supplementary Information

**Additional file 1.** Program on R package.

## Data Availability

The datasets used and/or analysed during the current study are available from the corresponding author and we can provide the sources (URLs/links) on reasonable request.

## Abbreviations

PB: Plastic bronchitis
FOB: Fiberoptic bronchoscopy
MP: *Mycoplasma pneumoniae*
CAP: Community-acquired pneumonia
LASSO: Least absolute shrinkage and selection operator
BCs: Bronchial casts
BAL: Bronchoalveolar lavage
PCR: Polymerase chain reaction
WBC: White blood cell
N%: Neutrophil ratio
L%: Lymphocyte ratio
PLT: Platelet counts
ESR: Erythrocyte sedimentation rate
CRP: C-reactive protein
PCT: Procalcitonin
IL-6: Interleukin-6
La: Lactic acid
LDH: Lactic dehydrogenase
ALT: Alanine aminotransferase
AST: Aspartate aminotransferase
CK: Creatine kinase
CK-MB: Creatine kinase isomer-MB
FER: Ferritin
APTT: Activated partial thromboplastin time
FG: Fibrinogen
CT: Chest computed tomography
IVIG: Intravenous immunoglobulin
ICU: Intensive care unit
MPP: *Mycoplasma pneumoniae* Pneumonia
RMPP: Refractory *Mycoplasma pneumoniae* pneumonia
AUC: Receiver operator characteristic curve
DCA: Decision curve analysis
